# Behavioral and Neural Manifestations of Reward Memory in Carriers of Low-Expressing versus High-Expressing Genetic Variants of the Dopamine D2 Receptor

**DOI:** 10.3389/fpsyg.2017.00654

**Published:** 2017-05-01

**Authors:** Anni Richter, Adriana Barman, Torsten Wüstenberg, Joram Soch, Denny Schanze, Anna Deibele, Gusalija Behnisch, Anne Assmann, Marieke Klein, Martin Zenker, Constanze Seidenbecher, Björn H. Schott

**Affiliations:** ^1^Leibniz Institute for NeurobiologyMagdeburg, Germany; ^2^Department of Psychiatry and Psychotherapy, Charité University HospitalBerlin, Germany; ^3^Institute of Human Genetics, Otto von Guericke UniversityMagdeburg, Germany; ^4^Department of Neurology, University of MagdeburgMagdeburg, Germany; ^5^Center for Behavioral Brain SciencesMagdeburg, Germany

**Keywords:** dopamine D2 receptor, TaqIA, C957T, episodic memory, reward, fMRI, intermediate phenotype

## Abstract

Dopamine is critically important in the neural manifestation of motivated behavior, and alterations in the human dopaminergic system have been implicated in the etiology of motivation-related psychiatric disorders, most prominently addiction. Patients with chronic addiction exhibit reduced dopamine D2 receptor (DRD2) availability in the striatum, and the *DRD2* TaqIA (rs1800497) and C957T (rs6277) genetic polymorphisms have previously been linked to individual differences in striatal dopamine metabolism and clinical risk for alcohol and nicotine dependence. Here, we investigated the hypothesis that the variants of these polymorphisms would show increased reward-related memory formation, which has previously been shown to jointly engage the mesolimbic dopaminergic system and the hippocampus, as a potential intermediate phenotype for addiction memory. To this end, we performed functional magnetic resonance imaging (fMRI) in 62 young, healthy individuals genotyped for *DRD2* TaqIA and C957T variants. Participants performed an incentive delay task, followed by a recognition memory task 24 h later. We observed effects of both genotypes on the overall recognition performance with carriers of low-expressing variants, namely TaqIA A1 carriers and C957T C homozygotes, showing better performance than the other genotype groups. In addition to the better memory performance, C957T C homozygotes also exhibited a response bias for cues predicting monetary reward. At the neural level, the C957T polymorphism was associated with a genotype-related modulation of right hippocampal and striatal fMRI responses predictive of subsequent recognition confidence for reward-predicting items. Our results indicate that genetic variations associated with DRD2 expression affect explicit memory, specifically for rewarded stimuli. We suggest that the relatively better memory for rewarded stimuli in carriers of low-expressing *DRD2* variants may reflect an intermediate phenotype of addiction memory.

## Introduction

Dopamine (DA) is crucially involved in motivated behavior, and dysfunctional dopaminergic neurotransmission has been implicated in the pathophysiology of neuropsychiatric disorders like schizophrenia and substance dependence (Heinz and Schlagenhauf, 2010). Therefore, numerous genetic association studies of these disorders have focused on the dopaminergic system. In humans, the *DRD2* gene on Chr 11q23.2, which encodes the dopamine D2 receptor, harbors several genetic variants previously linked to variability of D2 receptor expression as well as individual differences in motivated behavior and risk for psychiatric disorders. A common single nucleotide polymorphism (SNP, rs1800497; minor allele frequency 0.33 in dbSNP) located in the neighboring *ANKK1* gene, also known as TaqIA polymorphism, has repeatedly been linked to reduced striatal D2 receptor expression in A1 allele carriers in both *post mortem* expression investigations and *in vivo* radioligand binding studies using Positron emission tomography (PET; Noble et al., 1991; Thompson et al., 1997; Pohjalainen et al., 1998; Jonsson et al., 1999; Ritchie and Noble, 2003; Hirvonen et al., 2009a). One study employing single photon emission tomography (SPECT) did not find a difference in D2 receptor binding between A1 carriers and A2 homozygotes (Laruelle et al., 1998), but that study was later criticized for the combination of healthy participants and patients with schizophrenia in one sample and for the lower resolution of the SPECT compared to the PET method (Ritchie and Noble, 2003). The synonymous exonic *DRD2* C957T polymorphism (rs6277) has also been linked to striatal D2 receptor expression (Hirvonen et al., 2009a) and is in linkage disequilibrium (LD) with TaqIA (Ritchie and Noble, 2003; Doehring et al., 2009). Given the strong, but incomplete, LD between the polymorphisms, it is plausible to employ the haplotype defined by the two variants as a genetic proxy for D2 receptor expression (Gelernter et al., 2006; Doehring et al., 2009; Hirvonen et al., 2009a; Voisey et al., 2012). In clinical association studies, haplotypes containing both polymorphisms have been associated with impulsivity-related psychiatric disorders, most prominently addiction (Morton et al., 2006; Doehring et al., 2009; Voisey et al., 2012).

While the results of genetic association studies are thus far inconclusive with respect to actual disease risk (Samochowiec et al., 2014), studies of intermediate phenotypes have successfully demonstrated effects of genetic variability in the dopamine system on human motivational and cognitive processing (Meyer-Lindenberg and Weinberger, 2006; Yacubian et al., 2007; Richter et al., 2013, 2014; Wittmann et al., 2013). Most neurobiological investigations of addiction in humans and animals have highlighted the role of dysfunctional dopaminergic transmission in the ventral striatum/nucleus accumbens (NAcc) and reduced striatal D2 receptor availability (Kienast and Heinz, 2006; Everitt, 2014), but some authors have also pointed out the role of the hippocampus, which is critically involved in the formation of long-term memories (Robbins and Everitt, 2002; Robbins et al., 2008). Dopamine has been suggested to promote neural mechanisms underlying long-term memory formation and persistence, and this notion is supported by the previously reported preferential hippocampus-dependent encoding of reward-associated stimuli (Lisman and Grace, 2005; Wittmann et al., 2005; Adcock et al., 2006; Krebs et al., 2009; Lisman et al., 2011). With respect to genetic influences on reward memory, an imaging genetics study by Wittmann et al. (2013) has revealed that a genetic variation associated with dopamine transporter expression modulates the co-activation of the hippocampus and NAcc during the encoding of reward-associated information. Regarding potential effects of *DRD2* genetic variants on hippocampus-dependent memory, the C957T CC genotype has been associated with better episodic memory (Li et al., 2013; Papenberg et al., 2013), albeit studies on potential influences of the TaqIA polymorphism on explicit memory have yielded conflicting evidence. While Bartres-Faz et al. (2002) observed a protective effect of the A1 allele on long-term verbal memory performance among cognitively impaired elderly humans, Persson et al. (2015) found relatively lower memory performance in aged A1 carriers, particularly in tasks requiring verbal memory updating. Furthermore, McAllister et al. (2005, 2008) observed an adverse influence of the A1 allele on episodic memory for a word list in both healthy participants and patients with head injury. Importantly, none of those studies investigated a putative role of *DRD2* genetic variants on memory for reward-associated stimuli.

Bringing together the previous observations that the TaqIA A1 and C957T C alleles were associated with lower DRD2 expression, modulated neural processing in the striatum and limbic system, conferred a higher risk for substance abuse, and potentially affected episodic memory functions, we hypothesized that these genetic variants would influence hippocampus-dependent memory for reward-predicting stimuli. Specifically, we expected carriers of these variants to show increased reward-related memory and memory-related hippocampal and striatal activation as a potential intermediate phenotype for addiction memory. To this end, we performed functional magnetic resonance imaging (fMRI) during an incentive delay task followed by a delayed memory test (Wittmann et al., 2005, 2013; Barman et al., 2014, 2015) in a cohort of young healthy subjects genotyped for the TaqIA and the C957T polymorphisms. To investigate a potential influence of reward strength, we employed two reward conditions: a monetary and a social condition. In a previous study, we had found that the monetary condition elicited faster reaction times (RTs) and a more pronounced NAcc reward anticipation response than the social condition (Barman et al., 2015).

## Materials and Methods

The experimental paradigm, study cohort, data acquisition and fMRI data processing have been described previously in the context of another imaging genetics study and in a study on individual differences in autistic traits (Barman et al., 2014, 2015). In the first study, a modulation of the hippocampal recognition-encoding response for monetary reward-predicting items by a polymorphism of the guanine nucleotide exchange factor RASGRF1 was observed (Barman et al., 2014). The latter study investigated interactive effects of gender and subclinical autistic features on the anticipation and feedback processing of social reward (Barman et al., 2015). Here, we analyzed the memory parameters and their neural manifestation with respect to *DRD2* polymorphisms. Neither the inclusion of the RASGRF1 genotype nor of the Autism Quotient (AQ; Baron-Cohen et al., 2001) as covariates in our analysis leads to a qualitative change of the presented results.

### Participants

Sixty two participants (mean age ± SD: 24.58 ± 2.75 years) were recruited from a larger cohort of healthy and young volunteers of a large-scale behavioral genetics study conducted at the LIN Magdeburg (*n* = 719, age: mean ± SD = 23.77 ± 2.76 years; for detailed description of the sample see, Barman et al., 2014). Participants were recruited based on age, sex, and absence of MRI contraindications. All participants were of Caucasian origin, right-handed, had no history of neurological or psychiatric illness and did not use any illicit drugs or centrally acting medication according to self-report. Participants were stratified regarding the AQ to consider potential autistic traits as factor for social reward processing in a previous study (Barman et al., 2015).

Since TaqIA and C957T polymorphism have also been implicated in attentional processing and executive functions (Klein et al., 2007; Jocham et al., 2009; Markett et al., 2011; Colzato et al., 2013; Richter et al., 2013), the participants also performed standard neuropsychological tests like a flanker task (Eriksen and Eriksen, 1974), as well as the alertness and task switching subtests of the Test of Attentional Performance [*Testbatterie zur Aufmerksamkeitsprüfung* (TAP); Zimmermann and Fimm, 1993].

All participants gave written informed consent in accordance with the Declaration of Helsinki and received financial compensation for participation. The work was approved by the Ethics Committee of the University of Magdeburg, Faculty of Medicine.

### Genotyping

TaqIA polymorphism (NCBI accession number: rs1800497) was genotyped using a previously described protocol (Richter et al., 2013). Genomic DNA was extracted from blood leukocytes using the GeneMole^®^ automated DNA extraction system (Mole Genetics AS, Lysaker, Norway) according to the manufacturer’s protocol. Genotyping was performed using PCR with previously described primers (Grandy et al., 1989), followed by allele-specific restriction analysis with TaqI at 65°C.

For genotyping of C957T polymorphism (rs6277) a *Competitive Allele Specific PCR* assay (KASP; LGC Genomics GmbH, Berlin, Germany) was used. The reaction was performed in a final reaction volume of 10 μl containing 10 ng genomic DNA, 5 μl of 2x KASP Master mix (LGC Genomics) and 0.14 μl of primer mix with the two allele specific forward and the reverse primer. PCR-based amplification and read-out were performed in 96-well microtiter plates on the Roche LightCycler^®^ 480 Instrument II (Roche Diagnostics Germany, Mannheim, Germany) according to the manufacturer’s recommendation for this specific KOD assay. Data analysis was carried out using the LightCycler^®^ 480 Software release 1.5.0 (Roche Diagnostics Deutschland GmbH). Genotyping experiments were made with quality control of automated allele calling by two independent operators blinded to phenotype (100% concordance). The call rate for the genotyped marker was 100%.

Because the COMT Val108/158Met (rs4680), DAT1/VNTR (rs28363170), and RASGRF1 (rs8027411) polymorphisms have previously been linked to memory function and reward processing (Bertolino et al., 2006; Meyer-Lindenberg and Weinberger, 2006; Schott et al., 2006; Yacubian et al., 2007; Wittmann et al., 2013; Barman et al., 2014), participants were also genotyped for these polymorphisms (details available upon request).

### Procedure

We used a modified version of a previously employed categorical monetary incentive delay task conducted at two consecutive days (Wittmann et al., 2005; Krebs et al., 2009). On the first day participants performed a number comparison task in the MR tomograph. Besides monetary reward (1𝜀) participants could earn a positive social feedback (a photograph of smiling women, men, children, or couples) upon successful responding. Both reward types were investigated separately in two sessions. Each of the two sessions consisted of 100 trials (50 reward and 50 no-reward trials; event-related design), and the order of the runs was counterbalanced across participants. Before each session participants were given a short demonstration of the task and completed a practice session (20 trials) to learn the association between the cues and each condition. This practice session was employed to minimize learning effects during functional MR data acquisition and to induce the shift of the ventral striatal response from outcome to anticipation (Wittmann et al., 2005). Cue pictures consisted of photographs of simple objects that belonged to one of six categories (vehicles, bags, furniture, music instruments, clothes, kitchen devices). For each participant and session (monetary vs. social), two categories were randomly chosen to signal a potential reward or neutral outcome, respectively. Each trial started with the presentation of a cue picture for 1000 ms. Participants were asked to attend to the cues in order to be aware of the reward status and to respond via button press whether they expected a reward or not. After a variable delay (500–3500 ms), a number comparison task followed (target, 250 ms; Wittmann et al., 2005). Participants were requested to give a speeded response whether a target number was larger or smaller than five. The response deadline was adjusted individually based on RTs in the preceding trials to attain a correct response rate of approximately 80% (after four consecutive wins, the time limit was reduced by 20 ms, after one incorrect or slow reaction the time limit was increased by 20 ms). After a further variable time interval (500–2500 ms) a feedback was presented (750 ms). In reward trials either a picture of money coins or of a smiling face was presented upon fast and correct responses, and after a wrong or/and slow response black/white-noise image was shown. During neutral trials, the black/white-noise image was presented irrespective of outcome. The variable inter-trial interval was between 1000 and 4000 ms.

Twenty four (±4) hours after the start of the fMRI session, participants performed a recognition memory task outside the MR tomograph. Stimuli included the 200 cue images from the fMRI session, presented randomly intermixed with 100 distractors that had not been shown before. Subjects rated their recognition confidence on a scale ranging from 1 to 5 (“1”: definitely old; “2”: likely old; “3”: unsure; “4”: likely new; “5”: definitely new). These confidence ratings were used to model the relationship between successful encoding of the cue pictures and brain responses during the initial presentation of the pictures.

### MRI Data Acquisition

Functional MRI was performed using a 3 Tesla Siemens Magnetom Trio MR tomograph (SIEMENS Medical Systems, Erlangen, Germany) with a 12-channel phased array head coil. We collected structural (T1-weighted MPRAGE: 256 × 256 matrix; FOV = 256 mm; 96 2 mm sagittal slices) and functional images (Gradient-Echo echo-planar imaging [EPI] sequence; TR = 2000 ms; TE = 30 ms; FOV = 240 mm; flip-angle = 90°; matrix = 96 × 96; slice-thickness = 3 mm; 34 oblique slices parallel to the line from anterior to posterior commissure; voxel size = 2.5 mm × 2.5 mm × 3 mm; two runs of 420 volumes).

### fMRI Data Processing and Analysis

Image processing and statistical analyses were performed using Statistical Parametric Mapping (SPM12^1^). EPIs were corrected for acquisition time delay and head motion, spatially warped into the Montreal Neurological Institute (MNI) stereotactic reference frame, and spatially smoothed (isotropic Gaussian kernel; FWHM = 8 mm). A high-pass filter with a cut-off frequency of 128 s was applied to the data. Statistical analysis was carried out using a two-stage mixed-effects model. At the first stage, encoding-related hemodynamic responses were analyzed as a function of reward category-specific encoding and subsequent recognition confidence. Separate regressors for each reward category were created modeling the mean brain response. Recognition confidence-associated variance in brain responses was modeled by a trial-by-trial weighting of these regressors by the corresponding confidence ratings. Thus, the model contained eight regressors representing the memory-associated neural effects. Brain responses of no interest were modeled via regressors for targets and feedbacks, with the latter complemented by a parametric regressor for the feedback type (success/failure). Signal fluctuations caused by interactions of susceptibility and motion were modeled by means of the six rigid-body movement parameters determined from motion correction. Finally a constant regressor represented the signal mean of the time course. Model estimation was performed using a restricted maximum likelihood (ReML) fit as implemented in SPM. Since our research was focused on the effect of genotype on memory formation, linear contrasts of rewarded minus neutral trials were computed for the parametric modulated statistical maps for monetary and social reward categories separately. Thus, two linear contrast images per subject were submitted to second-level random-effects analyses of covariance (ANCOVA) with reward category (monetary vs. social) and genotype/haplotype (TaqIA: A1+ vs. A1-; C957T: CC vs. CT vs. TT; Haplotype: A1+/C+ vs. A1-/C+ vs. A1-/C-) as factors, and age and sex as covariates. Region of interest (ROI)-based analyses of recognition-encoding responses to reward-predicting items were performed using anatomical ROIs of the hippocampus (CA regions, as previously employed; Barman et al., 2014) generated with the SPM Anatomy Toolbox (Eickhoff et al., 2005) and of the striatum, generated with automated anatomical labeling (AAL; Tzourio-Mazoyer et al., 2002) implemented in the WFU-Pickatlas (Wake Forest University). Alpha error probabilities were adjusted for ROI-volumes [small volume correction (SVC)]. To this end, we first computed statistical maps with a significance level of *p* < 0.001 uncorrected and a minimum cluster size of 10 adjacent voxels. In a second step, the alpha errors for significant effects within the ROIs were corrected for the corresponding ROI-volume. Report and discussion was restricted to those findings with a resulting family-wise error (FWE) corrected alpha probability *p* < 0.05.

### Behavioral Data Analyses

To analyze the effects of motivation on the performance in the number comparison task, we calculated relative difference values between the RTs of correct responses in the neutral versus rewarded conditions divided by the mean RT for neutral trials, to account for confounding effects of individual variability of unspecific sensorimotor processing speed (Schott et al., 2007; DiffRT = [(RT_neutral_ – RT_reward_)/RT_neutral_^∗^ 100] for each subject. We then computed ANCOVAs with the genotype/haplotype as between-subject factor, reward type (monetary vs. social) as within-subject factor and age and sex as covariates.

To analyze the recognition of previously seen items, we calculated the corrected hit rate by subtracting the percentage of new items incorrectly judged as old (false alarms) from the percentage of correctly recognized old items (hits). To derive estimates of recollection and familiarity for each participant, receiver operating characteristics (ROCs) were generated by plotting the proportion of hits against the proportion of false alarms as a function of confidence and fit to a dual process model (Yonelinas et al., 2002; Duzel et al., 2011). The ANCOVAs were computed separately for each SNP and for the haplotype. All ANCOVAs included the genotype/haplotype (TaqIA: A1+ vs. A1-; C957T: CC vs. CT vs. TT; Haplotype: A1+/C+ vs. A1-/C+ vs. A1-/C-) as between-subject factor, trial type (rewarded vs. neutral), and reward category (monetary vs. social) as within-subject factors, and age and sex as covariates. When appropriate, correlational analyses (Pearson’s correlations), paired *t*-test or independent-sample *t*-test were used as *post hoc* tests.

To match the parametric modulation in the fMRI data analyses (see above), in an additional analysis, the medians of the Likert-scaled confidence ratings on the subsequent day were computed for each item type (old vs. new), trial type (rewarded vs. neutral) and reward category (social vs. monetary). Low values indicated that items were declared as old, and high values indicated that items were declared as new. To gain an initial all-encompassing overview including all within-subject factors, between-subject factors, and covariates, repeated-measures ANCOVAs were computed. Owing to the non-parametric nature of the dependent variable, non-parametric *post hoc* tests (Mann–Whitney-*U*, Kruskal–Wallis) were used to compare genotypes/haplotypes when appropriate. The ANCOVAs were computed separately for each SNP and for the haplotype. All ANCOVAs included the genotype or haplotype (TaqIA: A1+ vs. A1-; C957T: CC vs. CT vs. TT; Haplotype: A1+/C+ vs. A1-/C+ vs. A1-/C-) as between-subject factor, item type (old vs. new item), trial type (rewarded vs. neutral), and reward category (monetary vs. social) as within-subject factors, and age and sex as covariates.

## Results

### Genotyping

Among the 62 participants, we identified three A1 homozygotes, 28 heterozygotes, and 31 A2 homozygotes of the TaqIA polymorphism. A1 carriers (A1+: A1/A1 and A1/A2) were grouped together for all subsequent analyses and compared to A2 homozygotes (A1-: A2/A2) as in previous behavioral and imaging studies (Richter et al., 2013, 2014). The allelic distributions for the polymorphisms are displayed in **Tables 1–3**. The distribution of the SNPs did not violate Hardy–Weinberg equilibrium (HWE; TaqIA: χ^2^ = 1.12, *p* = 0.289; C957T: χ^2^ = 1.08, *p* = 0.300). As TaqIA and C957T are in LD, a combined analysis was conducted. Therefore we also grouped together C carriers of the C957T (C+: C/C and C/T; C-: T/T; Voisey et al., 2012), thus forming four possible haplotype combinations, of which only three were found in our cohort (**Table 3**). The groups of each genotype and the haplotype did not significantly differ in sex, age, allele distributions of the COMT Val108/158Met, RASGRF1 and DAT1/VNTR polymorphisms, smoking status or the AQ score (see **Tables 1–3**). Genotype/haplotype groups did also not differ in tests of attentional processes and executive functions (see Supplementary Tables S1–S3).

**Table 1:** Demographic data and behavioral data of the memory parameters regarding TaqIA polymorphism.

TaqIA	A1+	A2A2	Statistics
Women/Men	16/15	14/17	χ^2^ = 0.26, *p* = 0.611
Mean age	25.1 ± 3.3	24.0 ± 2.0	*t*_60_ = 1.61, *p* = 0.114
AQ	14.9 ± 7.0	15.4 ± 7.1	*U* = 464.00, *p* = 0.816
C957T CC/CT/TT	12/19/0	3/16/12	χ^2^ = 17.66, *p* < 0.001^∗^
COMT MM/VM/VV	6/22/3	8/14/9	χ^2^ = 5.06, *p* = 0.080
RASGRF1 GG/TG/TT	11/13/7	9/17/5	χ^2^ = 1.07, *p* = 0.587
DAT1 10-10/10-09/11-09	20/10/1	19/12/0	χ^2^ = 1.21, *p* = 0.547
Smoking status (no/yes)	19/11	26/5	χ^2^ = 3.32, *p* = 0.068

Corrected hit rates – monetary condition	
Neutral [%]	20.45 ± 12.50	20.26 ± 11.28	Main effect of TaqIA
Reward [%]	19.61 ± 12.06	15.61 ± 10.90	genotype
Corrected hit rates – social condition			*F*_1,58_ = 3.29, *p* = 0.075,
Neutral [%]	18.97 ± 14.18	18.45 ± 10.45	η^2^ = 0.05
Reward [%]	24.06 ± 14.32	18.19 ± 12.21	

Familiarity estimates – monetary condition	
Neutral	0.46 ± 0.35	0.39 ± 0.27	Main effect of TaqIA
Reward	0.45 ± 0.29	0.32 ± 0.26	genotype
Familiarity estimates – social condition			*F*_1,58_ = 4.10, *p* = 0.047^∗^,
Neutral	0.44 ± 0.39	0.39 ± 0.32	η^2^ = 0.06
Reward	0.43 ± 0.34	0.38 ± 0.32	

Median confidence ratings – monetary condition	
Neutral	3.50	3.50	Interaction of TaqIA
Reward	3.00	3.25	genotype × trial type ×
Median confidence ratings – social condition			reward category
Neutral	3.50	3.50	*F*_1,58_ = 2.89, *p* = 0.094,
Reward	3.50	3.50	η^2^ = 0.05

*Demographic data: sex distribution, age (years, mean *±* SD), AQ score (mean ± SD), number of smokers and non-smokers (data available for *N* = 61), and allelic distributions for C957T polymorphism, COMT Val108/158Met polymorphism (MM: Met homozygotes; VM: Val/Met heterozygotes; VV: Val homozygotes), RASGRF1 polymorphism and DAT1/VNTR polymorphism (10-10: 10 repeat homozygotes; 10-09: heterozygotes with 10 and 9 repeats; 11-09: heterozygotes with 11 and 9 repeats). Behavioral data: corrected hit rates (mean ± SD), familiarity estimates (mean ± SD), and median of confidence ratings. ^∗^Significant effect *p* < 0.005.*

**Table 2:** Demographic data and behavioral data of the memory parameters regarding C957T polymorphism.

C957T	CC	CT	TT	
Women/men	9/6	18/17	3/9	χ^2^ = 3.57, *p* = 0.168
Mean age	23.9 ± 2.3	25.1 ± 3.1	23.9 ± 1.8	*F*_2,59_ = 1.53, *p* = 0.226
AQ	15.6 ± 6.6	15.3 ± 7.6	14.2 ± 5.7	χ^2^ = 0.21, *p* = 0.901
COMT MM/VM/VV	3/10/2	7/22/6	4/4/4	χ^2^ = 3.94, *p* = 0.415
RASGRF1 GG/TG/TT	3/6/6	15/17/3	2/7/3	χ^2^ = 8.89, *p* = 0.064
DAT1 10-10/10-09/11-09	10/4/1	23/12/0	6/6/0	χ^2^ = 4.58, *p* = 0.333
Smoking status (no/yes)	12/2	23/12	10/2	χ^2^ = 2.77, *p* = 0.250

Corrected hit rates – monetary condition				
Neutral [%]	23.67 ± 11.68	18.69 ± 12.25	19.83 ± 10.04	Main effect of C957T
Reward [%]	20.13 ± 12.18	18.69 ± 10.98	11.33 ± 11.26	genotype
Corrected hit rates – social condition				*F*_2,57_ = 4.32, *p* = 0.018^∗^,
Neutral [%]	23.73 ± 12.58	17.49 ± 12.51	16.00 ± 10.69	η^2^ = 0.13
Reward [%]	26.27 ± 19.73	21.20 ± 9.61	14.50 ± 12.24	

Familiarity estimates – monetary condition				
Neutral	0.58 ± 0.33	0.35 ± 0.32	0.42 ± 0.21	Main effect of C957T
Reward	0.43 ± 0.33	0.40 ± 0.27	0.30 ± 0.26	genotype
Familiarity estimates – social condition				*F*_2,57_ = 3.75, *p* = 0.029^∗^,
Neutral	0.55 ± 0.44	0.35 ± 0.30	0.42 ± 0.36	η^2^ = 0.12
Reward	0.55 ± 0.45	0.39 ± 0.28	0.27 ± 0.25	

Median confidence ratings – monetary condition				
Neutral	3.50	3.50	3.00	Interaction of C957T
Reward	3.00	3.00	3.50	genotype × trial type ×
Median confidence ratings – social condition				reward category
Neutral	3.50	3.50	3.75	*F*_2,57_ = 4.37, *p* = 0.017^∗^,
Reward	3.00	3.50	3.50	η^2^ = 0.13

*Demographic data: sex distribution, age (years, mean ± SD), AQ score (mean ± SD), number of smokers and non-smokers (data available for *N* = 61), and allelic distributions for COMT Val108/158Met polymorphism (MM: Met homozygotes; VM: Val/Met heterozygotes; VV: Val homozygotes), RASGRF1 polymorphism and DAT1/VNTR polymorphism (10-10: 10 repeat homozygotes; 10-09: heterozygotes with 10 and 9 repeats; 11-09: heterozygotes with 11 and 9 repeats). Behavioral data: corrected hit rates (mean ± SD), familiarity estimates (mean ± SD), and median of confidence ratings. ^∗^Significant effect *p* < 0.005.*

**Table 3:** Demographic data and behavioral data of the memory parameters regarding the TaqIA/C957T haplotype.

Haplotype	A1+/C+	A1-/C+	A1-/C-	
Women/men	16/15	11/8	3/9	χ^2^ = 3.45, *p* = 0.179
Mean age	25.1 ± 3.3	24.1 ± 2.1	23.9 ± 1.8	*F*_2,59_ = 1.32, *p* = 0.275
AQ	14.9 ± 7.0	16.1 ± 7.9	14.2 ± 5.7	χ^2^ = 0.28, *p* = 0.868
COMT MM/VM/VV	6/22/3	4/10/5	4/4/4	χ^2^ = 6.21, *p* = 0.184
RASGRF1 GG/TG/TT	11/13/7	7/10/2	2/7/3	χ^2^ = 2.84, *p* = 0.585
DAT1 10-10/10-09/11-09	20/10/1	13/6/0	6/6/0	χ^2^ = 2.31, *p* = 0.679
Smoking status (no/yes)	19/11	16/3	10/2	χ^2^ = 3.33, *p* = 0.190

Corrected hit rates – monetary condition				
Neutral [%]	20.45 ± 12.50	20.53 ± 12.25	19.83 ± 10.04	Main effect of
Reward [%]	19.61 ± 12.06	18.32 ± 10.03	11.33 ± 11.26	haplotype
Corrected hit rates – social condition				*F*_2,57_ = 3.05, *p* = 0.055,
Neutral [%]	18.97 ± 14.12	20.00 ± 10.29	16.00 ± 10.69	η^2^ = 0.10
Reward [%]	24.06 ± 14.32	20.53 ± 11.92	14.50 ± 12.24	

Familiarity estimates – monetary condition				
Neutral	0.46 ± 0.35	0.37 ± 0.31	0.42 ± 0.21	Main effect of
Reward	0.45 ± 0.29	0.34 ± 0.27	0.30 ± 0.26	haplotype
Familiarity estimates – social cndition				*F*_2,57_ = 2.21, *p* = 0.119,
Neutral	0.44 ± 0.39	0.37 ± 0.29	0.42 ± 0.36	η^2^ = 0.07
Reward	0.43 ± 0.34	0.45 ± 0.35	0.27 ± 0.25	

Median confidence ratings – monetary condition				
Neutral	3.50	3.50	3.00	Interaction of
Reward	3.00	3.00	3.50	haplotype × trial type ×
Median confidence ratings – social condition				reward category
Neutral	3.50	3.50	3.75	*F*_2,57_ = 4.50, *p* = 0.015^∗^,
Reward	3.50	3.00	3.50	η^2^ = 0.14

*Demographic data: sex distribution, age (years, mean ± SD), AQ score (mean ± SD), number of smokers and non-smokers (data available for *N* = 61), and allelic distributions for COMT Val108/158Met polymorphism (MM: Met homozygotes; VM: Val/Met heterozygotes; VV: Val homozygotes), RASGRF1 polymorphism and DAT1/VNTR polymorphism (10-10: 10 repeat homozygotes; 10-09: heterozygotes with 10 and 9 repeats; 11-09: heterozygotes with 11 and 9 repeats). Behavioral data: corrected hit rates (mean ± SD), familiarity estimates (mean ± SD), and median of confidence ratings. ^∗^Significant effect *p* < 0.005.*

### Behavioral Results

#### Genotype/Haplotype-Related Modulation of Reward-Related Processing Speed

In the number comparison task, we observed a significant interaction of reward category and C957T genotype (*F*_2,57_ = 4.71, *p* = 0.013, η^2^ = 0.14), with C957T C carriers showing more reward-related RT decrease in the monetary versus social reward trials (CC: *t*_14_ = 6.62, *p* < 0.001; CT: *t*_34_ = 4.99, *p* < 0.001), while there was no difference for the TT homozygotes (*p* = 0.393). No further significant effects were observed (all *p* > 0.112).

#### Genotype/Haplotype-Related Modulation of the Corrected Hit Rates

**FIGURE 1:**
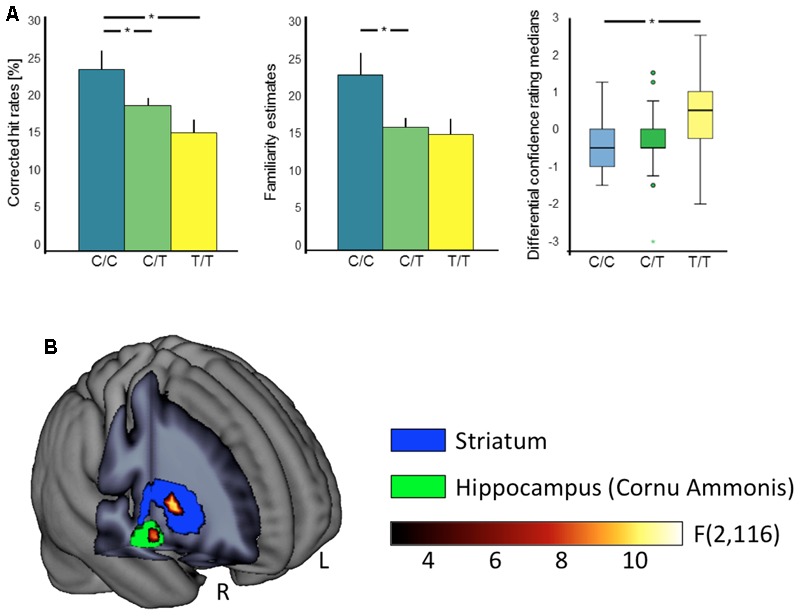
**Effects of the C957T polymorphism on behavioral and fMRI correlates of reward memory. (A)** Significant behavioral C957T genotype effects on episodic memory performance. Bar plots depict overall corrected hit rates (left) and familiarity estimates (middle) with standard errors. The C957T C allele is associated with an overall higher recognition performance. The box plot (right) depicts differential confidence rating medians (rewarded vs. neutral) in the monetary reward category. Horizontal lines represent the medians, the box represents the 25th and 75th percentiles, the whiskers indicate the 5th and 95th percentiles, dots mark outliers, and the colored asterisks indicate the extremes. Lower values of the C homozygotes indicate a bias to declare rewarded as compared to neutral items as old, independent of their actual item type. ^∗^*p* < 0.05. **(B)** Neural manifestations of the interaction between C957T genotype and reward category during encoding. Significant interactions of C957T genotype and reward category in right hippocampus (green) and striatum (blue). Statistical *F*-map and ROIs for small volume alpha error correction overlaid on a mean anatomical image. L, left; R, right.

With respect to delayed recognition, we observed a main effect of C957T genotype on the corrected hit rates, with trends in the same direction for TaqIA and for the haplotype (TaqIA: *p* = 0.075; C957T: *F*_2,57_ = 4.32, *p* = 0.018, η^2^ = 0.13; haplotype: *p* = 0.055; see **Tables 1–3**). C957T C homozygous subjects showed an overall better performance than heterozygous and T homozygous subjects (CC > CT: *t*_48_ = 2.10, *p* = 0.041; CC > TT: *t*_25_ = 2.60, *p* = 0.016; CT > TT: *p* = 0.083; see **Figure 1**).

Moreover there was a main effect of age in the model containing the haplotype (TaqIA: *F*_1,58_ = 4.29, *p* = 0.043, η^2^ = 0.07; C957T: *p* = 0.059; haplotype: *F*_1,57_ = 5.36, *p* = 0.024, η^2^ = 0.09), indicating a negative correlation between age and memory performance (*r* = -0.276, *p* = 0.030). All other effects or interactions were not significant (all *p* > 0.170).

#### Genotype/Haplotype-Related Modulation of Recollection and Familiarity Estimates

The analyses of the recollection estimates revealed no significant effects (all *p* > 0.066).

The analyses of the familiarity estimates revealed significant main effects of TaqIA and C957T genotype (TaqIA: *F*_1,58_ = 4.10, *p* = 0.047, η^2^ = 0.07; C957T: *F*_2,57_ = 3.75, *p* = 0.029, η^2^ = 0.12; haplotype: *p* = 0.119). TaqIA A1 carriers compared to A2 homozygotes (*t*_60_ = 1.48, *p* = 0.146; *t*-test with standardized residues accounting for age and sex: *t*_60_ = 2.03, *p* = 0.047), and C957T C homozygous subjects compared to heterozygous showed higher overall familiarity estimates (CC > CT: *t*_48_ = 2.12, *p* = 0.048). For the comparison between C957T C and T homozygous subjects we found only an – even equally directed – trend (CC > TT: *p* = 0.051). The comparioson between C957T heterocygotes and T homocygotes revealed no significant differences in familiarity (CT > TT: *p* = 0.690). A graphical depiction of these results could be found in **Figure 1**.

Again there was a main effect of age in the model containing the haplotype, and the TaqIA genotype (TaqIA: *F*_1,58_ = 5.83, *p* = 0.019, η^2^ = 0.09; C957T: *p* = 0.062; haplotype: *F*_1,57_ = 6.07, *p* = 0.017, η^2^ = 0.10), indicating a negative correlation between age and memory performance (*r* = -0.263, *p* = 0.039). Additionally an interaction of trial type (rewarded vs. neutral) and sex was observed in the model containing the TaqIA genotype (TaqIA: *F*_1,58_ = 4.21, *p* = 0.045, η^2^ = 0.07; C957T: *p* = 0.089; haplotype: *p* = 0.092), indicating slightly higher familiarity estimates in women compared to men in the rewarded, but not in the neutral condition (rewarded: *t*_60_ = 1.73, *p* = 0.089; neutral: *p* = 0.768). All further analyses yielded no significant effects (all *p* > 0.069).

#### Genotype/Haplotype-Related Modulation of Reward-Related Recognition Confidence Ratings

When analyzing the medians of the confidence ratings, all three ANCOVAs revealed a main effect of the item type (all *p* < 0.029), indicating that previously seen items were indeed recognized as old (lower medians for old vs. new items).

Moreover we observed significant three-way interactions of C957T genotype and haplotype with trial type and reward category (TaqIA: *p* = 0.094; C957T: *F*_2,57_ = 4.37, *p* = 0.017, η^2^ = 0.13; haplotype: *F*_2,57_ = 4.49, *p* = 0.015, η^2^ = 0.14), most notably, in absence of an interaction with item type (four-way interaction: all *p* > 0.435). *Post hoc* tests showed that in the monetary reward category, differential confidence ratings (rewarded vs. neutral) in C975T C homozygotes versus T homozygotes revealed a bias to declare rewarded items as old, independent of their actual item type (old vs. new; CC > TT: monetary: *U* = 40.50, *p* = 0.015, social: *p* = 0.500; see **Figure 1**). A1+/C+ haplotype carriers compared to A1-/C- haplotype carriers (A1+/C+ > A1-/C-: monetary: *U* = 99.50, *p* = 0.018, social: *p* = 0.377; see **Tables 2, 3** for details) also showed significant differences of confidence ratings, but as no effect was observed for the A+/C+ haplotype carriers compared to A1-/C+ haplotype carriers (A1+/C+ > A1-/C+: monetary: *p* = 0.959, social: *p* = 0.760), this effect was most likely driven by LD with the C957T polymorphism.

In the model containing the TaqIA genotype, on the other hand, a significant three-way interaction of trial type × reward category × sex was observed (*F*_1,58_ = 5.09, *p* = 0.028, η^2^ = 0.08), most likely reflecting a response bias of men in the social and of women in the monetary category (men > women: monetary: *U* = 315.50, *p* = 0.018; social: *U* = 335.00, *p* = 0.037).

All other effects were not significant (all *p* > 0.077).

### Brain Activation Patterns

#### Genotype/Haplotype-Related Modulation of Neural Correlates of Reward-Dependent Memory

To investigate the neural correlates of reward-related memory, we modeled recognition confidence-associated variance in brain responses via linear parametric regressors (see Materials and Methods). Reward category-specific contrast images between reward and neutral trials were submitted to group level ANCOVA. We observed significant interactions of C957T genotype and reward category in right hippocampus and striatum (right hippocampus: *F*_2,116_ = 8.80, *p* = 0.032, [x y z] = [24 -13 -14], 9 voxels; right striatum: *F*_2,116_ = 10.43, *p* = 0.026, [x y z] = [30 5 1], 14 voxels; *p* < 0.05, FWE-corrected for the respective ROI volumes; see **Figure 1**), indicating a C957T-related modulation of hippocampal and striatal correlates of reward-dependent memory.

## Discussion

Our results show a genotype-dependent modulation of hippocampal and striatal brain responses during encoding of reward-predicting items. Importantly, this modulation manifested also at the behavioral level, with genotypes previously linked to lower D2 receptor expression, i.e., the TaqIA A1 and C957T C alleles, being associated with an overall higher recognition performance and a response bias for reward-predicting items.

### Dopamine D2 Receptor Gene Variants and Recognition Performance

In the present study, we observed significant effects of both the *DRD2* C957T and the *DRD2/ANKK1* TaqIA gene variants on recognition memory, with carriers of the low-expressing alleles showing higher corrected hit rates and familiarity estimates. Our findings are compatible with two previous studies demonstrating relatively superior memory performance in C957T C homozygotes (Li et al., 2013; Papenberg et al., 2013). Previous results regarding the TaqIA polymorphism have been inconsistent (see Introduction; Bartres-Faz et al., 2002; McAllister et al., 2005, 2008; Persson et al., 2015). Effects of TaqIA might have been driven by its LD with C957T, but none of the previous studies investigated both SNPs together, nor have those studies tested memory for reward-associated stimuli. In the present study, we had hypothesized that DRD2 genotypes would preferentially affect the encoding of picture stimuli that predicted a reward. While we found low-expressing DRD2 variants to be associated with overall memory performance and with a more liberal response criterion for rewarded stimuli, we did not observe a specific interaction of genotype and reward on the actual memory performance at the behavioral level. On the other hand, such an interaction was observed at the level of memory-related brain activity. The most likely explanation for this is, in our view, the relatively small sample size. It has previously been suggested that differences in BOLD signal changes are likely to be more closely related to the cellular effects mediated by genetic variations than the between-group differences of behavioral readouts (Meyer-Lindenberg and Weinberger, 2006; Mier et al., 2010).

A further somewhat unexpected finding was that genotype-related differences in memory performance were found for familiarity, but not recollection estimates. This observation is to some extent in contrast to the previously reported higher recollection rates for reward-predicting items (Wittmann et al., 2005). One explanation for this discrepancy comes from modeling work by Elfman et al. (2008) who postulate that recollection versus familiarity in explicit memory processes is influenced by item similarity. Specifically, as the level of feature similarity across items increases, the hippocampus loses its ability to encode items distinctively, and the threshold nature of recollection – as opposed to familiarity, which follows signal detection theory – breaks down. In line with this explanation, the stimuli used in the original study by Wittmann et al. (2005) were considerably less similar to each other, and the categories were more broadly defined (living vs. non-living objects).

### Dopaminergic Modulation of Hippocampus-Dependent Memory Formation

Our data analyses revealed that, in addition to effects of both the *DRD2/ANKK1* TaqIA and the C957T genotypes on recognition memory, the C957T polymorphism also modulated hippocampal and striatal activation during encoding of reward-predicting stimuli. The hippocampus and the striatum, particularly the NAcc, are core structures of a neural circuit that has been suggested to mediate the encoding of novel and reward-associated information into long-term memory, the so-called hippocampal-VTA loop (Lisman and Grace, 2005). According to this model, dopamine release in the hippocampus and NAcc promotes long-term memory by stabilizing plasticity mechanisms, which may underlie the well-documented superior memory performance for rewarded relative to unrewarded stimuli (Wittmann et al., 2005; Adcock et al., 2006; Krebs et al., 2009).

It may seem counterintuitive that individuals carrying genetic variations associated with lower striatal D2 receptor density exhibit better reward-related memory. It should be noted, though, that higher baseline dopaminergic tone, as indexed by PET imaging of dopamine synthesis capacity has been linked to detrimental rather than beneficial effects of reward on attentional performance (Aarts et al., 2014). With respect to memory, a similar observation has been reported in participants who performed a recognition memory task with a reward manipulation. Reward affected recognition performance adversely when participants had received the dopamine precursor L-DOPA (Apitz and Bunzeck, 2013). Both studies convergingly support the previously suggested inverted U-shape of dopaminergic effects on human cognitive processing (Vijayraghavan et al., 2007).

An additional or possibly alternative explanation for the observed pattern might be a potential role of extrastriatal D2 receptors in reward memory. It should be noted though, that D2 receptor expression outside the striatum is sparse and constitutes to a considerable degree of presynaptic inhibitory autoreceptors (for reviews see Wolf and Roth, 1990; Schmitz et al., 2003). With respect to the TaqIA polymorphism, lower expression of autoinhibitory D2 receptors has been proposed to elicit increased presynaptic dopamine synthesis (Laakso et al., 2005), which may conceivably also influence extrastriatal dopamine release. While this notion is compatible with both animal studies (Bello et al., 2011; de Jong et al., 2015), and a pharmacological study in humans (Buckholtz et al., 2010). Along the same line Wittmann et al. (2013) observed a modulation of striatal and hippocampal activation during successful encoding of reward-related pictures by a polymorphism previously associated with striatal dopamine transporter expression (meta-analysis Costa et al., 2011; Shumay et al., 2011; Spencer et al., 2013) and presumably resulting extracellular DA availability. Compatibly, reward circuit activity has been linked to interindividual variability of striatal dopamine release (Schott et al., 2008), and increased midbrain and NAcc activity in nine-repeat carriers has also been observed during successful episodic memory formation, independently of reward (Schott et al., 2006). However, the existing data regarding C957T on striatal versus extrastriatal D2 receptor binding are thus far inconclusive (Hirvonen et al., 2009a,b; see Limitations and Directions for Future Research), and it seems therefore premature to simply attribute the observed association of C957T with reward memory to reduced extrastriatal presynaptic autoinhibition.

### Response Bias as a Further Risk Mechanism for Addiction Memory?

Our data analyses revealed that, in addition to overall better reward memory performance, C957T C homozygotes exhibited also a response bias for rewarded items. That is, that the analysis of the medians of the confidence ratings of the recognition test 24 h after encoding revealed that carriers of low-expressing alleles showed a tendency to judge images that predicted monetary reward as old, irrespective of whether they had actually been presented during encoding. The observation that this bias was only apparent in the monetary and not the social condition may reflect the stronger propensity of the monetary condition to elicit reward responses (Barman et al., 2015). High false alarm rates can be induced experimentally, for example by a well-known paradigm described by Roediger and McDermott, in which the context of a lure item -that is not actually presented during study- is induced, leading to increased false recognition of the lure item at test (Roediger and McDermott, 1995). While the original finding by Roediger and McDermott has been replicated by a number of different groups, the underlying neurocognitive mechanisms are not yet completely understood, and it is unlikely that a single process leads to the increased recognition of the lures (Jou and Flores, 2013). In the original paradigm, lure items were typically category words, while a number of associate words were presented during study. In the present study, carriers of low-expressing *DRD2* alleles showed a tendency to judge items from the monetary reward category as old, even though these items did not differ qualitatively from other images of the same category. One mechanism that has been proposed to underlie the tendency to judge new items as old is a shift of response criterion (Miller and Wolford, 1999). Such a criterion shift could also happen when an entire category is more salient than another. Given the previously reported increased risk of substance-related disorders in carriers of low-expressing *DRD2* alleles (Morton et al., 2006; Doehring et al., 2009; Swagell et al., 2012; Voisey et al., 2012; also underpinned by a nominal TaqIA genotype effect on the smoking status in our cohort, see **Table 1**), the higher false alarm rate could also be considered a tendency to generalize reward-associated stimuli and to show a reduced ability to inhibit a response to such stimuli (see, for example, Machulska et al., 2016). This interpretation is also compatible with our previous observation in a motivated Go/Nogo learning task, in which TaqIA A1 carriers showed a selective deficit in learning the “NoGo-to-win” condition, i.e., the suppression of a motor response to obtain a reward (Richter et al., 2014). While these studies cannot elucidate the precise molecular mechanisms, they may nevertheless deliver potential intermediate phenotypes.

### Limitations and Directions for Future Research

The most important limitation of the present study is the relatively small sample size, which is the most likely reason why no significant interaction effects of genotype and reward on memory performance could be observed (see above). Furthermore, given that genetic variations do not exert their effects in isolation, it would be of interest to assess potential interactions with other gene variants in the dopamine system. For example Wittmann et al. (2013) observed a modulation of striatal and hippocampal activation during successful encoding of reward-related pictures by the DAT1/VNTR polymorphism, and in our own group we observed effects of a polymorphism of the guanine nucleotide exchange factor RASGRF1 that is an important regulator of intracellular signaling and neural plasticity in the brain (Barman et al., 2014). Another potential variant of interest would be the COMT Val108/158Met (rs4680) polymorphism that has been previously associated with both memory function and reward processing (Bertolino et al., 2006; Meyer-Lindenberg and Weinberger, 2006; Schott et al., 2006; Yacubian et al., 2007). The sample size, however, did not allow us to systematically investigate such combined genetic effects. We did, however, test the allelic distribution of those variants in order to exclude them as potential confounds.

Another limitation, albeit not unique to the present study, is that the exact effects of C957T on dopamine D2 receptor availability are yet incompletely understood. While *in vitro* studies have suggested lower mRNA stability associated with the T allele (Duan et al., 2003), an *in vivo* PET investigation has demonstrated that, unexpectedly, C homozygotes had the lowest striatal D2 receptor binding potential (Hirvonen et al., 2004). In a follow-up study, Hirvonen et al. (2009a) further suggested that this effect was better attributable to receptor affinity rather than actual expression levels. For extrastriatal D2 receptors, one study has actually suggested increased rather than decreased binding potential in C homozygotes (Hirvonen et al., 2009b), which would, in case of presynaptic D2 receptors, be in conflict with our interpretation that lower extrastriatal presynaptic DRD2 expression might result in higher activity of the hippocampal-VTA loop in C957T C carriers. On the other hand, Hirvonen et al. (2009b) suggested that their results could be rather attributable to the – generally sparsely expressed – post-synaptic extrastriatal D2 receptors, as D2 autoreceptor functioning in the cortex was less efficient compared to the striatum (Cubeddu et al., 1990), and the cortical expression pattern might thus more likely reflect the regulations at mRNA level described by Duan et al. (2003). It should further be noted that the lifelong presence of a genetic variant associated with altered gene expression or regulation is likely to lead to long-term plasticity at the large-scale network level. With respect to the DRD2 C957T polymorphism, for example, Markett et al. (2013) have reported reduced striatal gray matter density in C homozygotes, which may in turn lead to long-term changes in cortico-striatal loop function, thereby exerting subtle effects on cognitive functions like memory.

## Conclusion

Our results provide evidence for a role of *DRD2*-SNPs in human reward memory with carriers of low-expressing *DRD2* alleles being associated with an overall higher recognition confidence and a response bias for reward-predicting items. This pattern may reflect a phenomenon contributing to a complex endophenotype, which at a clinical level manifests as addiction memory (see also Robbins et al., 2008) and reward-related impulsivity.

## Author Contributions

Designed experiment: AR, AB, AD, JS, BS. Performed experiment: AB, DS, AD, GB, AA, MK, MZ. Analyzed data: AR, AB, TW, JS. Wrote manuscript: AR, DS, CS, BS. All authors edited and/or approved the manuscript.

## Conflict of Interest Statement

The authors declare that the research was conducted in the absence of any commercial or financial relationships that could be construed as a potential conflict of interest.

## Footnotes

1 http://www.fil.ion.ucl.ac.uk/spm/
