# Supplementary material for: Behavioral and Neural Manifestations of Reward Memory in Carriers of Low-Expressing versus High-Expressing Genetic Variants of the Dopamine D2 Receptor
Source: Front Psychol. 2017 May 1;8:654. doi: 10.3389/fpsyg.2017.00654 (PMC5410587; doi:10.3389/fpsyg.2017.00654)
Supplement: Supplementary file 1 [file Table_1.docx]

**Supplementary Material**

**Table S1:** Neuropsychological characterization of the sample regarding TaqIA polymorphism

| **TaqIA** | **A1+** | **A2A2** | **Statistics** |
| --- | --- | --- | --- |
| Flanker task |  |  |  |
| *Flanker effect RT [ms]* | 109 ± 70 | 141 ± 91 | *t*_60_ = -1.57, *p* = .122 |
| *Flanker effect errors [%]* | 7.6 ± 8.3 | 11.5 ± 12 | *t*_60_ = -1.51, *p* = .137 |
| Alertness |  |  |  |
| *Tonic alertness [ms]* | 270 ± 28 | 266 ± 30 | *F*_1,59_ = 0.98, *p* = .327 |
| *Phasic alertness [ms]* | 240 ± 41 | 228 ± 38 |  |
| Task Switching |  |  |  |
| *Correct switching [%]* | 97.6 ± 2.9 | 96.5 ± 4.4 | *F*_1,60_ = 0.45, *p* = .506 |
| *Correct no-switching [%]* | 93.8 ± 7.6 | 93.1 ± 8.3 |  |
| *RT switching [ms]* | 1100 ± 284 | 1106 ± 230 | *F*_1,60_ = 0.02, *p* = .904 |
| *RT no-switching [ms]* | 1280 ± 345 | 1258 ± 245 |  |

Behavioral data (Mean ± SD) of a flanker task, a test for alertness (data available for N = 62) and a test for task switching. RT: reaction time of correct responses.

**Table S2:** Neuropsychological characterization of the sample regarding C957T polymorphism

| **C957T** | **CC** | **CT** | | **TT** |  |
| --- | --- | --- | --- | --- | --- |
| Flanker task |  | |  | |  |
| *Flanker effect RT [ms]* | 104 ± 50 | 132 ± 89 | | 131 ± 95 | *F*_2,59_ = 0.65, *p* = .526 |
| *Flanker effect errors [%]* | 6.9 ± 8.4 | 10.0 ± 10.4 | | 11.7 ± 12.9 | *F*_2,59_ = 0.75, *p* = .479 |
| Alertness |  | |  | |  |
| *Tonic alertness [ms]* | 261 ± 16 | 272 ± 33 | | 265 ± 28 | *F*_2,58_ = 1.35, *p* = .268 |
| *Phasic alertness [ms]* | 232 ± 31 | 240 ± 45 | | 218 ± 32 |  |
| Task Switching |  | |  | |  |
| *Correct switching [%]* | 97.8 ± 2.0 | 96.8 ± 4.5 | | 97.0 ± 3.2 | *F*_2,59_ = 0.24, *p* = .786 |
| *Correct no-switching [%]* | 94.3 ± 6,0 | 92,9 ± 9.1 | | 94.3 ± 6.8 |  |
| *RT switching [ms]* | 1086 ± 223 | 1117 ± 282 | | 1085 ± 223 | *F*_2,59_ = 0.09, *p* = .912 |
| *RT no-switching [ms]* | 1235 ± 245 | 1269 ± 321 | | 1311 ± 301 |  |

Behavioral data (Mean ± SD) of a flanker task, a test for alertness (data available for N = 62) and a test for task switching. RT: reaction time of correct responses.

**Table S3:** Neuropsychological characterization of the sample regarding the TaqIA/C957T haplotype

| **Haplotype** | **A1+/C+** | **A1-/C+** | | **A1-/C-** |  |
| --- | --- | --- | --- | --- | --- |
| Flanker task |  | |  | |  |
| *Flanker effect RT [ms]* | 109 ± 70 | 148 ± 91 | | 131 ± 95 | *F*_2,59_ = 1.36, *p* = .264 |
| *Flanker effect errors [%]* | 7.6 ± 8.3 | 11.4 ± 11.9 | | 11.7 ± 12.9 | *F*_2,59_ = 1.16, *p* = .334 |
| Alertness |  | |  | |  |
| *Tonic alertness [ms]* | 270 ± 28 | 267 ± 32 | | 265 ± 28 | *F*_2,58_ = 0.86, *p* = .431 |
| *Phasic alertness [ms]* | 240 ± 41 | 235 ± 41 | | 218 ± 32 |  |
| Task Switching |  | |  | |  |
| *Correct switching [%]* | 97.6 ± 2.8 | 96.2 ± 5.2 | | 97.0 ± 3.2 | *F*_2,59_ = 0.46, *p* = .635 |
| *Correct no-switching [%]* | 93.9 ± 7.5 | 92.3 ± 9.2 | | 94.3 ± 6.8 |  |
| *RT switching [ms]* | 1100 ± 284 | 1120 ± 240 | | 1085 ± 223 | *F*_2,59_ = 0.04, *p* = .959 |
| *RT no-switching [ms]* | 1280± 345 | 1224 ± 204 | | 1311 ± 301 |  |

Behavioral data (Mean ± SD) of a flanker task, a test for alertness (data available for N = 62) and a test for task switching. RT: reaction time of correct responses.
